# The validity and reliability of the Arabic version of the EQ-5D in atrial fibrillation patients in a conflict country: a study from Syria

**DOI:** 10.1186/s12872-024-04203-4

**Published:** 2024-10-08

**Authors:** Ibrahim Antoun, Alkassem Alkhayer, Majed Aljabal, Alamer Alkhayer, Peter Simon, Yaman Mahfoud, Ahmed Kotb, Joseph Barker, Akash Mavilakandy, Riyaz Somani, G Andre Ng, Mustafa Zakkar

**Affiliations:** 1 Department of Cardiovascular Sciences, Clinical Science Wing, Glenfield Hospital, Leicester, UK; 2 Faculty of Medicine, University of Aleppo, Aleppo, Syria; 3 University of Tishreen’s Hospital, Latakia, Syria; 4 Leicestershire Partnership NHS Trust, Leicester, UK; 5 NIHR Leicester Biomedical Research Centre, Leicester, UK; 6 National Heart and Lung Institute, Imperial College London, London, UK; 7 Department of Cardiology, University Hospitals of Leicester NHS Trust, Glenfield Hospital, Leicester, UK; 8 Department of Cardiac Surgery, University Hospitals of Leicester NHS Trust, Glenfield Hospital, Leicester, UK; 9 Faculty of Medicine, University of Damascus, Damascus, Syria; 10 Department of Cardiovascular Sciences, Glenfield Hospital, Groby Road, Leicester, LE3 9QP UK

**Keywords:** Quality of life, Syria, Atrial fibrillation, Conflict, EQ-5D

## Abstract

**Background:**

The EQ-5D is one of the most commonly used tools to establish health-related quality of life (QoL). EQ-5D data in atrial fibrillation (AF) patients in the Middle East are lacking.

**Objectives:**

This study aims to evaluate the reliability and validity of the Arabic version of the EQ-5D in AF inpatients in Syria.

**Methods:**

The study involved patients admitted to the emergency department of Tishreen’s University Hospital in Latakia with AF as the primary diagnosis between the 1st of June 2021 and the 1st of June 2023. Arabic versions of the EQ-5D, EQ-VAS and SF36 questionnaires were administered to patients. Validation was done using convergent, discriminant, and known-groups validity, while reliability was conducted using EQ-5D retesting within 2–4 weeks.

**Results:**

432 participants were included in the study with a mean ± standard deviation of 63 ± 15. Males represented 242 (56%) of the participants. All hypotheses relating EQ-5D responses to external variables were satisfied. All three validation hypotheses demonstrated that the EQ-5D had the convergent, discriminant and known group validity to assess QoL in this cohort. The intraclass correlation coefficient (ICC) for test-retest reliability ranged between 0.74 and 0.88, while Cohen’s κ ranged between 0.72 and 0.86. Cronbach’s α value for internal consistency was 0.73.

**Conclusion:**

The Arabic version of EQ-5D was valid and reliable in measuring QoL in AF inpatients in Syria. This validation opens the door for more widespread use of the EQ-5D in Arabic-speaking regions, facilitating better-informed healthcare decisions and improving patient care strategies in Syria and other Middle Eastern countries.

## Background

Health-related quality of life (QoL) is a self-perceived well-being related to or affected by disease or treatment [1]. In Syria (an Arabic-speaking country), conducting clinical trials is challenging, and QoL studies are mainly done with Syrian refugees abroad [2, 3]. The EQ-5D is a standardised generic instrument used to measure health and QoL outcomes. It is available and validated in most significant languages with cultural adaptations. It provides an index value and a simple descriptive profile for the health status [4]. It applies to various health conditions and treatments, including cardiovascular conditions such as atrial fibrillation (AF) [5, 6]. Understanding the psychometric properties in similar countries like Iran is crucial for ensuring its validity, reliability, and applicability in these contexts. For example, two studies from Iran validated the EQ-5D-3 L and EQ-5D-5 L in diabetes mellitus and cancer, respectively [7, 8].

Furthermore, the different diseases can be evaluated differently. For example, studies from Iran suggest that the EQ-5D-5 L provides better sensitivity and validity in complex health conditions such as cancer. At the same time, the EQ-5D-3 L is helpful in chronic conditions like diabetes but may benefit from disease-specific assessments [7, 8]. These insights are crucial for accurate health assessments in similar contexts. AF is the most common sustained arrhythmia, and its prevalence in low to middle-income countries is likely underestimated [9]. Although AF in the Western world is extensively studied, little data corroborates AF demographics and management in the Middle East, with only four epidemiological data registries [10]. AF-related research is still insufficient in the Arab world, contributing only 0.7% of the total AF research [11]. AF is well known to impair QoL amongst various questionnaires [12]. However, there is little data regarding QoL in the Arab world, nor known QoL questionnaires were validated to do so for AF patients in these countries. One of these countries, Syria, has been in conflict since 2011 and deprived of healthcare resources and funding, particularly exacerbated during the COVID-19 and cholera outbreaks [13, 14]. As a result, less than half of its hospitals operate at full performance, with over 50% of its healthcare workforce forced to leave due to conflict. More than half the Syrian population live in poverty, which is suggested to increase AF risk [15, 16].

Furthermore, two recent Syrian studies demonstrated challenges in managing inpatient AF patients with high readmission rates [17, 18]. Therefore, the QoL of AF patients in Syria is a critical concern, given the country’s complex healthcare and social context. By conducting comprehensive assessments through tailored questionnaires, we can gain valuable insights that will inform healthcare policies, interventions, and support systems, ultimately improving the lives of AF patients in Syria.

As there has been a call to validate the EQ-5D in cardiovascular conditions in the Middle East [19], this study aims to validate the EQ-5D questionnaire that has already been validated in neighbouring Arabic countries [15, 16] to contribute to better AF patient-centred and effective healthcare strategies for this vulnerable population.

## Methods

The study group was a convenience sample of consecutive Arabic-speaking patients > 18 years without an apparent cognitive deficit who were admitted through the emergency department of Tishreen’s University Hospital and treated for AF as the primary diagnosis between the 1st of June 2021 and the 1st of June 2023. Secondary AF patients were excluded. Patients in high-dependency units and intensive care were also excluded. Tishreen’s University Hospital is Latakia’s largest hospital, providing free health care to the population and is a cardiology tertiary centre. Medical students with prior experience with patient interactions through clinical rotations in various medical disciplines conducted the interviews using the EQ-5D and SF-36 in their fifth and sixth years of medical education. Before conducting the interviews, the students underwent specific training on administering the EQ-5D and SF-36 instruments. This training included detailed sessions on the purpose and structure of the surveys, interview techniques, ethical considerations, and mock interviews with standardised patients. Senior clinicians and researchers supervised the training to ensure reliability and accuracy in administering the questionnaires. The questionnaire contained the Arabic EQ-5D version and the short-form health survey (SF-36-Arabic version) [20]. A random sample was picked, given a copy of the EQ-5D and re-interviewed using the EQ-5D after two to four weeks over the phone to assess test-retest reliability. Demographics and socioeconomic status were obtained during the questionnaire.

This study was part of a quality improvement project at the institution, and patients provided informed consent. The hospital board and the ethical committee at Tishreen’s University Hospital approved the study (Trial reference number: 277/A).

### EQ-5D and SF-36 questionnaires

The EQ-5D is a preference-based instrument used to assess QoL. Although translated into multiple languages, it has been translated and culturally adapted into Arabic in several countries, including Jordan and Saudi Arabia [15, 16]. The EQ-5D includes two parts: a classification system of five dimensions (mobility, usual activities, self-care, anxiety/depression and pain/discomfort) and a EuroQol Visual Analogue Scale (EQ-VAS). The EQ-VAS is a straight line ranging from 0 (representing the worst imaginable health) to 100 (representing the best imaginable health), allowing individuals to define their current health status.

The SF-36 questionnaire has been extensively used and validated in several Arabic-speaking countries, such as Lebanon [21]. Research has demonstrated that the Arabic version of the SF-36 is dependable and accurate for evaluating QoL across diverse populations. It includes 36 questions divided into eight scales: physical functioning, bodily pain, role-physical, general health, vitality, role-emotional, social functioning, and mental health. The eight scales are summarised into a mental and physical component [22].

### Statistical analysis

Continuous variables were expressed as mean and standard deviation or median and standard deviation (SD). Categorical variables were expressed as counts and percentages (%). Pearson’s χ 2 or Fisher’s exact test was used for categorical variables between groups. Mann-Whitney U or student t-tests compared continuous variables between the groups depending on the normal distribution of the data decided by the D’Agostino-Pearson normality test.

We hypothesise that the EQ-5D is reliable and valid in assessing QoL in Syrian patients admitted with AF.

### Reliability

An article by Kennedy stated that sample sizes of at least 100 are required to establish Cronbach’s α [23]. Cronbach’s α values investigated the internal consistency of the EQ-5D scales, giving a score between 0 and 1, a value of > 0.70 suggesting good internal consistency [24]. Test-retest reliability was measured using intraclass correlation coefficient (ICC). We consider ICC values lower than 0.5, between 0.5 and 0.75, between 0.75 and 0.9 and higher than 0.9, respectively, indicative of poor, moderate, good and excellent reliability [25]. Test-retest reliability was also assessed using Cohen’s Kappa. Cohen’s guidelines for interpreting correlation coefficients were used; correlations between 0.50 and 1.00 were interpreted as strong, correlations between 0.30 and 0.50 as moderate, correlations between 0.10 and 0.30 as small and correlations < 0.1 as weak [26].

### Validity

The construct validity was assessed using convergent, discriminant, and known-groups validity. Convergent validity was measured through Spearman’s correlation coefficient between the EQ-5D dimensions, EQ-VAS scores and the SF-36 scales. We expected the degree of correlation between the dimensions of EQ-5D that are theoretically very similar to scales of SF-36 to be higher than those of the EQ-5D dimensions for which SF-36 are theoretically dissimilar (for example, the mobility dimension of the EQ-5D is expected to have a higher correlation with physical function compared to the emotional function of the SF-36) [7, 27]. To test the discriminant validity of the EQ-5D, we compared the average scores of the SF-36 scale between patients who reported no problems or mild problems (Level 1) and those who reported moderate (Level 2) or severe (Level 3) problems in each dimension of the EQ-5D [7]. We used a t-test to compare the means. We expected the average SF-36 scores for patients reporting no problems to be higher than those with moderate or severe problems [7].

The known-group validity of the mean of combined EQ-5D and EQ-VAS scores was assessed by testing the difference between their score for each characteristic of patients using an independent sample t-test. The mean QoL score of the EQ-5D and EQ-VAS was expected to be better QoL (lower mean EQ-5D and higher mean EQ-VAS) in male patients, younger patients, more educated, employed, and patients without chronic disease [28–30]. A significance level of *P* < 0.05 was agreed. Statistical analysis was performed using SPSS Statistics for Windows, Version 29.0. Armonk, NY: IBM Corp.

## Results

596 adult patients presented to the hospital with a primary diagnosis of AF. All of them speak and understand Arabic. Of those, 74 were not in a clinical state to conduct the questionnaire, 24 declined to participate, and 66 had missing EQ-5D data sets, which were excluded from the analysis, leaving 432 participants. Table 1 demonstrates the subjects’ demographics and socioeconomic status. Table 2 shows the response distribution to EQ-5D dimensions. Anxiety was the most prevalent major problem in 161 participants (37%).

**Table 1:** General characteristics of study subjects stated by patients on admission

Factor	Value
Age (years)	63 ± 15
Male	242 (56%)
Participants with chronic disease	186 (43%)
Education	
Not educated	60 (14%)
Primary	86 (20%)
Secondary	197 (45%)
University	89 (21%)
Marital status	
Single	74 (17%)
Married	285 (67%)
Widowed	73 (16%)
Employment	
Employed	144 (33%)
Unemployed	110 (25%)
Housewife	80 (19%)
Retired	98 (23%)

**Table 2:** EQ-5D response distribution across study subjects

	Mobility	Self-care	Activities	Pain	Anxiety
No Problem	43 (10%)	92 (21%)	82 (19%)	50 (12%)	39 (9%)
Moderate problem	137 (32%)	81 (19%)	94 (22%)	113 (26%)	120 (28%)
Severe problem	104 (24%)	143 (33%)	109 (25%)	124 (29%)	161 (37%)

### Convergent validity

The results are demonstrated in Table 3. The range of correlation between the SF-36 scales and EQ-5D included − 0.29 to -0.77 in mobility, -0.4 to -0.57 in self-care, -0.39 to -0.85 in usual activities, -0.39 to -0.84 in pain/discomfort and − 0.32 to -0.91 in anxiety/depression. The correlation between mobility and physical component summary was stronger than between mobility and mental component summary (-0.47 versus − 0.33). On the other hand, the correlation between anxiety and the mental component summary was stronger than between anxiety and the physical component summary (-0.59 versus − 0.39). The range of correlation between the EQ-VAS scores and SF-36 scales was 0.84–0.93.

**Table 3:** Correlation between the EQ-5D dimensions, EQ-VAS scores and SF-36 scales

	EQ-5D dimensions					EQ-VAS
	Mobility	Self-care	Activities	Pain	Anxiety	
Physical functioning	-0.77*	-0.43*	-0.56*	-0.59*	-0.32*	0.91*
Role physical	-0.38*	-0.57*	-0.56*	-0.79*	-0.32*	0.93*
Bodily pain	-0.38*	-0.52*	-0.85*	-0.84*	-0.37*	0.84*
General health	-0.43*	-0.56*	-0.59*	-0.58*	-0.48*	0.91*
Vitality	-0.39*	-0.51*	-0.58*	-0.57*	-0.46*	0.9*
Social functioning	-0.37*	-0.53*	-0.5*	-0.51*	-0.47*	0.91*
Role-emotional	-0.29*	-0.43*	-0.39*	-0.39*	-0.38*	0.81*
Mental health	-0.33*	-0.4*	-0.45*	-0.42*	-0.91*	0.95*
Physical component summary	-0.47*	-0.52*	-0.63*	-0.70*	-0.39*	0.90*
Mental component summary	-0.33*	-0.45*	-0.45*	-0.44*	-0.59*	0.89*

Spearman’s rank correlation coefficient was used

*p < 0.001

## Discriminant validity

Table 4 presents the results of the EQ-5D’s discriminatory ability between different levels of health according to SF-36. As expected, level 1 in each EQ-5D dimension was associated with higher scores on all SF-36 scales. All differences between level 1 and level 2 or 3 in each of the EQ-5D dimensions were significant.

**Table 4:** The SF-36 scales by dimension and level of the EQ-5D

		Physical functioning	Role physical	Bodily pain	General health	Vitality	Social functioning	Role-emotional	Mental health
Mobility	1	60 (26)*	60 (28)*	70 (35)*	70 (34)*	60 (26)*	60 (33)*	50 (25)*	55 (27)*
	2	38 (20)*	40 (28)*	52 (34)*	50 (35)*	48 (23)*	40 (27)*	46 (28)*	40 (26)*
	3	13 (0*	34 (26)*	38 (28)*	28 (36)*	34 (27)*	34 (21)*	34 (17)*	34 (24)*
Self-care	1	51 (28)*	61 (29) *	70 (34)*	72 (32)*	66 (23)*	62 (30)*	58 (26)*	58 (28)*
	2	40 (24)*	48 (25)*	52 (35)*	47 (27)*	48 (24)*	41 (21)*	40 (24)*	40 (25)*
	3	14 (7)*	24 (17)*	30 (19)*	14 (8)*	22 (9)*	24 (16)*	26 (15)*	30 (16)*
Usual activities	1	63 (30)*	83 (30)*	83 (32)*	86 (21)*	75 (21)*	76 (24)*	66 (27)*	72 (26)*
	2	37 (24)*	43 (23)*	45 (30)*	41 (24)*	46 (23)*	34 (29)*	47 (22)*	42 (27)*
	3	18 (11)*	27 (21)*	30 (20)*	19 (11)*	25 (16)*	25 (17)*	24 (15)*	30 (13)*
Pain/discomfort	1	54 (28)*	69 (25)*	89 (21)*	76 (29)*	67 (23)*	66 (29)*	57 (25)*	57 (27)*
	2	35 (20)*	50 (19)*	42 (13)*	42 (25)*	44 (23)*	35 (20)*	46 (22)*	47 (23)*
	3	16 (12)*	15 (12)*	21 (15)*	11 (8)*	19 (7)*	24 (15)*	13 (8)*	20 (12)*
Anxiety/ depression	1	50 (31)*	61 (32)*	71 (34)*	74 (30)*	66 (23)*	63 (29)*	80 (14)*	76 (22)*
	2	43 (23)*	50 (26)*	56 (33)*	52 (36)*	50 (25)*	46 (31)*	41 (6)*	46 (12)*
	3	29 (21)*	36 (25)*	41 (31)*	31 (26)*	36 (26)*	32 (20)*	20 (11)*	21 (13)*

*p < 0.01 for t-test. Dimensions are in mean (standard deviation)

## Known-groups validity

Table 5 shows that males, employed, educated, and patients without chronic disease have a better quality of life, characterised by a lower mean EQ-5D and a higher EQ-VAS score.

**Table 5:** Comparison of the mean of the EQ-5D and the EQ VAS scores according to patient characteristics

Variable	*n* (%)	EQ-5D, mean of all dimensions (SD)	EQ-VAS, mean (SD)
**Gender**			
Female	190 (44%)	3 (1)^*^	39 (26) ^*^
Male	242 (56%)	2.3 (1) ^*^	58 (25) ^*^
**Age**			
≥ 60	250 (58%)	3 (0.97) ^*^	37 (25) ^*^
< 60	182 (42%)	2.3 (1) ^*^	60 (25) ^*^
**Education**			
Not educated	60 (14%)	3.2 (1) ^*^	35 (22) ^*^
Primary education or above	372 (86%)	2.2 (1) ^*^	63 (27) ^*^
**Employed**			
Not employed, retired or housewife	288 (67%)	3.4 (1.1) ^*^	31 (20) ^*^
Employed	144 (33%)	2 (0.8) ^*^	68 (21) ^*^
**Chronic disease other than AF**			
Yes	186 (43%)	3.5 (1) ^*^	29 (16) ^*^
No	246 (57%)	1.9 (0.8) ^*^	69 (22) ^*^

*p < 0.05 using t-test

### Reliability

Cronbach’s α value was 0.73 and 0.79 for EQ-5D and EQ-VAS, suggesting that both questionnaires have an acceptable level of internal consistency in this Syrian cohort. The Arabic EQ-5D was administered twice to 40 respondents (30%), with a median follow-up time of 4 weeks (interquartile range: 3–5 weeks). The ICC for the five EQ-5D dimensions were 0.79, 0.78, 0.74, 0.82, and 0.88 for, self-care, usual activities, mobility, pain/discomfort, and anxiety/depression, respectively (*P* < 0.001 for all dimensions). Similarly, Cohen’s κ values for the five EQ-5D dimensions were 0.77, 0.76, 0.72, 0.79, and 0.86 for self-care, usual activities, mobility, pain/discomfort, and anxiety/depression, respectively (*P* < 0.001 for all dimensions). ICC and Cohen’s κ for the EQ-VAS were 0.75 and 0.71, respectively (*P* < 0.001).

## Discussion

Multiple studies focused on the validity and reliability of the EQ-5D questionnaire in Europe [31] and the Middle East [16, 19]. However, this is the first study to establish the validity and reliability of the EQ-5D instrument in the Syrian population and the first study to confirm the validity and reliability of the EQ-5D questionnaire in an Arabic-speaking AF cohort. All hypotheses linking the Arabic EQ-5D to SF-36 were satisfied, suggesting that the Arabic EQ-5D has characteristics comparable to those of other EQ-5D versions. This was in keeping with the Jordanian and the Saudi Arabian data [16, 19]. the questionnaire’s internal consistency was acceptable, and the test-retest reliability of the EQ-5D self-classifier, with ICC being moderate to good (0.74–0.88) and Cohen’s κ being strong to excellent (0.71–0.86). Also, the significant and high intraclass correlation coefficient obtained in the EQ-VAS reliability study suggests that it is a feasible measure of self-reported health in this Syrian AF population. This is in keeping with studies in neighbouring countries, including Saudi Arabia [15] and Jordan [16], which demonstrated that the EQ-5D is valid and reliable in assessing QoL in the Arabic population. Comparing our results to data from non-Arabic-speaking countries with similar socioeconomic status, two Iranian studies validating the score for diabetes mellitus and cancer showed similar ICC, ranging from 0.66 to 0.92 [7, 8]. However, our Cohen’s κ was superior with a narrower range than the Iranian (0.39–0.71 versus 0.71–0.86). However, Cronbach’s alpha was higher than our study (0.88 versus 0.73). Future studies should aim to focus on testing the reliability and validity of these questionnaires amongst variable diseases in Syria and comparing them to similar diseases in similar socioeconomic countries.

Assessing QoL in AF patients in Syria helps understand the specific challenges and needs of these patients. It could inform policy decisions to improve their overall health outcomes and QoL in challenging circumstances. It recognises that healthcare decisions and treatments should be tailored to the individual’s preferences and needs. QoL assessments provide a holistic view of a patient’s well-being beyond clinical measures, enabling healthcare providers to address their unique concerns and goals. This aligns with the principles of patient-centred care proposed in the latest European Society of Cardiology guidelines [32]. This can contribute to the body of knowledge regarding the epidemiology of the condition. Research findings can help identify patterns, risk factors, and effective interventions relevant to the Syrian population. This would enormously help AF management in Syria, along with other conditions if proven valid, given the ongoing conflict and scarcity of funding.

A strength of this study is its use of widely used instruments to measure QoL among Arabic-speaking AF patients, which is widely spoken worldwide. Another important outcome of this study was its value in exploring and validating the Arabic EQ-5D through testing several hypotheses that compare responses to EQ-5D dimensions or EQ-VAS to SF-36 scores. This finding is precious and sets the stage for utilising this important tool for measuring QoL among AF patients in numerous Middle Eastern countries. This also encourages using EQ-5D in other conditions in the Syrian population as a cost-effective tool to measure outcomes.

One study limitation was that we used a convenience sample that only included AF inpatients, which may limit the generalizability of the results. This limitation was evident as a high level of education characterised the sample. Another limitation is that there was no approved QoL questionnaire against which to compare the EQ-5D. The study only focused on specific factors influencing QoL. It did not explore other potential factors that could have an impact, such as unmeasured biopsychosocial factors or the dynamic interplay between them. These unexplored factors might affect how applicable the findings are to the general population [33, 34]. However, it’s important to note that this study is just a pilot investigation, and we plan to conduct a more extensive study that includes a more representative sample of the population.

## Conclusion

The Arabic EQ-5D was valid and reliable in evaluating QoL in Syrian AF patients admitted to the hospital. This suggests that the instrument is well-suited for capturing the health-related QoL in this specific patient population, providing a robust framework for assessing patient outcomes in clinical settings. The study underscores the importance of linguistically and culturally adapted tools in health research, ensuring that the measurements are accurate and relevant across different populations. This validation opens the door for more widespread use of the EQ-5D in Arabic-speaking regions, facilitating better-informed healthcare decisions and improving patient care strategies in countries undergoing conflict, such as Syria.

## Data Availability

No datasets were generated or analysed during the current study. Data relating to this study are available upon reasonable request from the corresponding author.
